# Integrative Transcriptomic Analysis Identifies COL3A1 as a Potential Tumor-Intrinsic Therapeutic Target in NSCLC

**DOI:** 10.3390/biomedicines14050975

**Published:** 2026-04-23

**Authors:** Kaicheng Zhou, Yanyang Nan, Mengyang Li, Dongyue Hou, Caili Xu, Haiyan Yu, Jun Feng, Dianwen Ju, Ziyu Wang

**Affiliations:** 1 School of Pharmaceutical Sciences, Shanghai Engineering Research Center of Immunotherapeutics, Fudan University, Shanghai 201203, China; 2 Novatim Immune Therapeutics (Zhejiang) Co., Ltd., Huzhou 313300, China; 3 National Key Laboratory of Lead Druggability Research, China State Institute of Pharmaceutical Industry Co., Ltd., Shanghai 201203, China; 4 Shanghai Duomirui Bio-tech Co., Ltd., Shanghai 201203, China; 5 Department of Pharmacy, Huadong Hospital, Fudan University, Shanghai 200040, China

**Keywords:** non-small cell lung cancer, single-cell transcriptomic, programmed death ligand-1, epithelial–mesenchymal transition, collagen type III alpha 1

## Abstract

**Background:** Non-small cell lung cancer (NSCLC) remains a leading cause of cancer-related mortality, and although PD-1/PD-L1 immune checkpoint blockade has improved outcomes in some patients, therapeutic responses remain heterogeneous. Tumor-intrinsic heterogeneity within malignant epithelial populations is increasingly recognized as a critical determinant of disease progression and therapy response. **Methods:** Here, we constructed a comprehensive single-cell atlas of NSCLC by integrating 650,461 cells from 216 tumor and normal samples. Tumor-derived epithelial cells were reclustered to identify transcriptionally distinct subpopulations. Pseudotime analysis, functional experiments, and in vivo validation using a humanized xenograft model were performed to investigate the role of COL3A1. **Results:** Reclustering of tumor-derived epithelial cells revealed 25 transcriptionally distinct subpopulations. Among these, a high-risk cluster exhibited coordinated activation of epithelial–mesenchymal transition (EMT) and angiogenesis programs and was associated with poor patient survival. Within this aggressive subpopulation, Collagen type III alpha 1 (COL3A1) emerged as a tumor-intrinsic gene associated with extracellular matrix remodeling and angiogenic signaling. Pseudotime analysis indicated that COL3A1^+^ cells represent a late-stage, poorly differentiated malignant state. Functional experiments demonstrated that COL3A1 knockdown impaired NSCLC cell proliferation, migration, and invasion. Virtual knockout further suggested that COL3A1 may be associated with transcriptional programs involved in PD-L1 upstream signaling pathways, indicating a potential indirect link between tumor-intrinsic states and immune regulatory networks. Consistently, in vivo silencing of COL3A1 enhanced the antitumor efficacy of PD-L1 blockade. **Conclusions:** Collectively, our study identifies COL3A1 as a tumor-intrinsic gene enriched in malignant epithelial cells with mesenchymal features and a potential therapeutic target. These findings provide a rationale for exploring combinatorial strategies integrating tumor-intrinsic pathway inhibition with immune checkpoint blockade in NSCLC.

## 1. Introduction

Non-small cell lung cancer (NSCLC) remains the leading cause of cancer-related deaths worldwide [1,2]. Although immune checkpoint inhibitors targeting the PD-1/PD-L1 axis have improved outcomes in some patients, responses are highly variable, and intrinsic resistance remains common [3,4]. Increasing evidence suggests that tumor-intrinsic transcriptional programs and heterogeneity within malignant epithelial populations critically influence disease progression and therapy response.

Recent advances in single-cell RNA sequencing (scRNA-seq) have enabled high-resolution mapping of tumor ecosystems, revealing substantial heterogeneity among malignant epithelial cells [5,6]. Distinct tumor cell states can differ in proliferative capacity, metastatic potential, angiogenic activity, and interactions with the immune microenvironment. However, the molecular drivers and evolutionary trajectories that shape aggressive epithelial states in NSCLC remain incompletely understood. In particular, how epithelial plasticity, angiogenic reprogramming, and immune modulation are interconnected warrants further investigation [7].

Epithelial–mesenchymal transition (EMT) and angiogenesis are central hallmarks of tumor progression and have been increasingly recognized as contributors to immune evasion and resistance to immunotherapy [8]. Blood vessel formation not only facilitates nutrient supply and metastatic dissemination but also creates immunosuppressive niches within tumors. Identifying tumor-intrinsic regulators that coordinately drive these programs in specific malignant subpopulations is therefore crucial [9].

Collagen type III alpha 1 chain (COL3A1) is a major structural component of the extracellular matrix, previously implicated in tissue remodeling, tumor invasion, and metastasis across multiple cancers, including lung, breast, and colorectal cancers [10,11,12]. While prior studies primarily examined COL3A1 expression at the bulk-tissue level or within stromal compartments, its distribution and functional role within specific malignant epithelial subpopulations remain poorly understood. Using single-cell transcriptomic analyses, we found that COL3A1 is highly enriched in a late-stage, poorly differentiated epithelial subpopulation in NSCLC characterized by coordinated activation of EMT and angiogenesis programs. This tumor-intrinsic expression suggests that COL3A1 may be involved in extracellular matrix remodeling and is associated with aggressive malignant phenotypes, vascular reprogramming, and potentially immune checkpoint-related signaling, providing a rationale for its investigation as a cell-state-specific therapeutic target.

In this study, we constructed a comprehensive single-cell atlas of NSCLC to systematically characterize malignant epithelial heterogeneity. Through integrated CNV inference, trajectory analysis, survival deconvolution, and multi-model prognostic validation, we identified a clinically relevant aggressive epithelial state characterized by coordinated activation of EMT and angiogenesis programs. Within this subpopulation, COL3A1 emerged as a tumor-intrinsic regulator. Using computational perturbation, in vitro functional assays, and in vivo combination therapy experiments, we show that COL3A1 is associated with malignant phenotypes and may be linked to transcriptional programs related to PD-L1 signaling, providing a mechanistic rationale for combined therapeutic strategies in NSCLC.

## 2. Experimental Procedures

### 2.1. Data Availability

Single-cell RNA-seq data (raw data) for NSCLC were obtained from GEO and EMBL-EBI. Bulk RNA-seq data and corresponding clinical data were acquired from TCGA-LUAD and TCGA-LUSC cohorts. All datasets are publicly available and no additional ethical approvals were required. In total, scRNA-seq datasets were included, comprising both tumor and normal lung samples, with detailed sample composition and sequencing depth as follows: EMTAB6149 (15 tumor and 3 normal samples; 50,616 cells), EMTAB6653 (9 tumor and 2 normal samples; 27,945 cells), GSE117570 (4 tumor and 3 normal samples; 11,276 cells), GSE123902 (8 tumor and 4 normal samples; 33,038 cells), GSE127465 (18 tumor samples; 65,400 cells), GSE131907 (15 tumor and 11 normal samples; 95,433 cells), GSE148071 (42 tumor samples; 75,697 cells), GSE148466 (3 tumor samples; 3918 cells), GSE189357 (7 tumor samples; 116,555 cells), PRJNA591860 (49 tumor samples; 20,474 cells), PRJNA634159 (11 tumor samples; 127,136 cells), and PRJNA698465 (12 tumor samples; 22,973 cells).

### 2.2. scRNA-Seq Data Processing

Initially, CellRanger (v6.0.0) was implemented for preprocessing wherever accessible sequencing data existed in a raw, unprocessed format [13]. These datasets underwent transformation to FASTQ files, which subsequently allowed alignment against the refdata-gex-GRCh38-2020-A human genomic reference by means of the CellRanger count workflow. As a result of this analytical pipeline, gene expression count matrices were efficiently generated.

Downstream single-cell analyses were based on R (v4.4.2) using Seurat (v5.1.0) [6]. Cells were excluded if they expressed fewer than 200 genes, had fewer than 500 detected RNA features, or contained > 10% mitochondrial transcripts. Gene expression values were normalized using LogNormalize with a scaling factor of 10,000. The top 2000 highly variable genes were identified using FindVariableFeatures and scaled using ScaleData.

Moving forward, principal component analysis (PCA) enabled reduction in dimensionality, leveraging the 30 most significant principal components to address batch-related heterogeneity through correction with Harmony (v1.2.1) [14]. To delineate cellular subsets, the FindNeighbors and FindClusters methods were employed, whereas underlying structure was visualized using UMAP projections for spatial and compositional interpretations.

To systematically classify individual cellular populations, markers characteristic of specific lineages were applied as guidance. Hallmark genes provided robust signatures: epithelial cell states reflected by EPCAM, KRT8, KRT18; fibroblastic element demarcated with COL1A1, COL1A2, FAP; endothelial fractions characterized using VWF, CDH5, PECAM1; lymphocyte lineages annotated via CD3D, CD3E for T lymphocytes and CD79A, MS4A1 for B cells; innate cytotoxic clusters assigned using NKG7, GNLY, KLRD1 to trace natural killer cell differentiation; myeloid components, including macrophages and monocytes, distinguished by expression of CD68, S100A8, S100A9, and CD163 [15].

### 2.3. Copy Number Variation (CNV) Analysis

To identify neoplastic epithelial cells relative to their non-neoplastic counterparts, inferCNV version 1.20.0 was employed, with epithelial cells derived from neighboring unaffected samples serving as the baseline reference group [16]. Genes localized on the sex chromosomes or within mitochondrial DNA regions were omitted before proceeding with computations. The estimation of copy number variation was achieved with denoising activated and a threshold set to 0.1, and analysis was executed without the application of a hidden Markov model algorithm.

Relative CNV scores were calculated based on signal variance per cell using the complete set of epithelial cells and normalized to reference epithelial cells. Subpopulations were classified as Tumor-like or Normal-like based on relative CNV intensity. For visualization purposes only, subsampling was applied (maximum of 200 tumor cells per subcluster and 2000 reference epithelial cells) to ensure successful heatmap generation and avoid excessive memory usage during inferCNV plotting.

### 2.4. TCGA Bulk RNA-Seq Processing

Bulk RNA-seq data from TCGA-LUAD and TCGA-LUSC were downloaded using TCGAbiolinks (v2.32.0) [17]. Only primary tumor samples were retained. TPM expression values were transformed as log2(TPM + 1).

Ensembl gene identifiers were converted to HGNC gene symbols using biomaRt (v2.60.1) [18]. Genes without valid annotation were removed. For duplicated gene symbols, expression values were averaged.

Overall survival (OS) time was defined as days to death or days to last follow-up. Samples with missing survival information were excluded. Survival time ≤ 0 was adjusted to 0.1 days. Clinical variables include age, sex, and TNM stage.

### 2.5. Bulk RNA-Seq Deconvolution and Survival Analysis

BayesPrism (v2.2.2) was applied to estimate tumor epithelial subpopulation proportions in TCGA samples using single-cell tumor cells as reference [19]. Mitochondrial, ribosomal, sex chromosome, and low-expression genes were removed prior to deconvolution.

Proportions for each cell-type estimated from bulk RNA-seq analysis were consolidated with clinical survival datasets. Kaplan–Meier survival analysis was conducted utilizing the ‘survival’ (v3.7-0) as well as ‘survminer’ (v0.5.0) R packages. Patient cohorts were categorized as high or low according to whether their subpopulation fraction exceeded the group median. To evaluate differences in overall lifespan, restricted mean survival time (RMST) was calculated using the survRM2 tool (version 1.0-4) over a span of 3650 days [20].

### 2.6. Pathway Enrichment and Scoring

For tumor-versus-normal comparisons, pseudo-bulk matrices were constructed by averaging gene expression within each Celltype × tissue group. ssGSEA was performed using GSVA (v1.52.3)with Hallmark gene sets retrieved via msigdbr (v7.5.1) [21,22]. Differential pathway activity was calculated as Δscore = Tumor − Normal.

For single-cell pathway scoring, Hallmark pathway scores were calculated by averaging log-normalized expression of pathway genes per cell. Scores were integrated into Seurat metadata and compared across tumor subpopulations using Wilcoxon rank-sum tests.

### 2.7. Differential Expression and Key Gene Selection

Cluster-specific marker genes were identified using FindMarkers, which set min.pct equal 0.25. Genes were ranked by average log2 fold change. The top 10 upregulated genes in Cluster 20 were intersected with Hallmark EMT and angiogenesis gene sets to identify candidate regulators. Overlaps were visualized using Venn diagrams.

### 2.8. Expression Density Visualization

Gene expression density was visualized using Nebulosa (v1.14.0), applying kernel density estimation on UMAP embeddings to highlight spatial enrichment patterns [23].

### 2.9. Trajectory and Pseudotime Analysis

Tumor epithelial cells were analyzed using Monocle (v2.32.0) [24]. Highly variable genes were used as ordering genes. A CellDataSet object was constructed from raw count matrices. Dimensionality reduction and cell ordering were performed using DDRTree. Cells were categorized as epithelial-like, COL3A1^+^, or COL3A1^−^ based on expression levels for visualization purposes. CytoTRACE (v0.3.3) was applied to assess differentiation potential and validate trajectory direction [25]. Branch Expression Analysis Modeling (BEAM) was used to identify branch-dependent genes (q ≤ 0.01). Genes were clustered into four modules using ClusterGVis (v0.1.4), followed by GO Biological Process enrichment analysis for each module [26].

### 2.10. Survival Modeling and Machine Learning Analysis

Univariate and multivariate Cox proportional hazards regression analyses were performed using the survival R package (v3.7-0). Patients were dichotomized by median COL3A1 expression for Kaplan–Meier survival analysis.

Random survival forest (RSF) modeling was conducted using randomForestSRC (v3.4.5). Variable importance was calculated using permutation-based VIMP. Partial dependence plots were generated to assess nonlinear associations between COL3A1 expression and predicted mortality risk. Patients were stratified by RSF-derived risk scores for survival comparison.

XGBoost survival modeling was performed using xgboost (v3.1.2.1) under the Cox objective function. Feature importance was evaluated using Gain and SHAP metrics. SHAP dependence plots were generated to assess marginal contributions of COL3A1 expression to predicted survival risk.

### 2.11. Virtual Knockout and Pathway Analysis

Virtual gene knockout analysis was performed in Cluster 20 tumor cells using scTenifoldKnk (v1.0.3) [27]. Regulatory pathways were inferred before and after COL3A1 perturbation. Differentially regulated genes were ranked by perturbation strength, excluding COL3A1 itself. The top 40 genes were selected for downstream analysis.

Reactome pathway enrichment was performed on the top 40 genes [28]. Global transcriptional changes were visualized using volcano plots. Gene set enrichment analysis (GSEA) was conducted using Reactome gene sets, and pathways with NES > 1.5 and *p* < 0.05 were considered significant.

### 2.12. Cell Culture

NSCLC cell lines H1975 and A549, sourced from the Type Culture Collection of the Chinese Academy of Sciences in Shanghai, China, were propagated in RPMI 1640 medium (Gibco, Thermo Fisher Scientific, Waltham, MA, USA; Catalog No. 11875119) containing 10% fetal bovine serum (Gibco, Thermo Fisher Scientific, Waltham, MA, USA; Catalog No. 10099141) under a humidified atmosphere with 5% CO_2_ at 37 °C.

### 2.13. Cell Proliferation Assay (CCK-8)

The assessment of cell proliferation employed the Cell Counting Kit-8 (CCK-8; Meilunbio, Dalian, China; Catalog No. MA0218). Cells transfected with the relevant siRNAs were placed into 96-well plates, 2000 cells per well. CCK-8 reagent was introduced to each sample well at different intervals, specifically on days one through three, with plates incubated at 37 °C for sixty minutes. Absorbance readings at 450 nm were performed using a microplate reader, and triplicate samples’ optical density data were used to plot growth curves.

### 2.14. siRNA Transfection

Small interfering RNAs (siRNAs) targeting COL3A1 (si-COL3A1, sense: 5′-GGACAAAGGUGACAGAGGA-3′) and negative control siRNA (siNC, sense: 5′-UUCUCCGAACGUGUCACGU-3′) were synthesized by RiboBio (Guangzhou, China). Prior to transfection, NSCLC cells were distributed into six-well plates to ensure 70–90% cell confluency after 24 h. Transfections adhered strictly to protocols detailed by the manufacturer, utilizing Lipofectamine 3000 (Invitrogen, Carlsbad, CA, USA; Catalog No. L3000015). SiRNAs and Lipofectamine reagent, prepared independently in Opti-MEM medium, were merged and permitted to incubate at room temperature for ten to fifteen minutes prior to their administration to cells. Following a twenty-four-hour period, cells were gathered for further verification of COL3A1 suppression by means of Western blotting.

### 2.15. Transwell Migration and Invasion Assays

The migration and invasion potential of cells was analyzed utilizing 24-well Transwell inserts equipped with 8 µm pores (Corning, NY, USA; Catalog No. 3422). For migration assessment, 5 × 10^4^ transfected cells suspended in a basal medium lacking serum were placed in the upper compartment, while DMEM augmented with 20% fetal bovine serum filled the lower chamber. Following 24 h incubation, migratory cells were subjected to methanol fixation and 1% crystal violet staining before enumerating cells in three randomly selected microscopic fields. In invasion testing, prior to cell seeding, Matrigel matrix (BD Biosciences, San Jose, CA, USA; Catalog No. 356234) diluted 1:8 in serum-free medium was distributed across the upper insert, permitting solidification at 37 °C. All subsequent processing steps mirrored those used for cell migration assays. The total duration from siRNA transfection to the end of the assay was 48 h, and COL3A1 knockdown was confirmed by Western blot at the assay endpoint.

### 2.16. Western Blot Analysis

Cells were lysed in RIPA buffer (Beyotime, Shanghai, China; Catalog No. P0013B) supplemented with protease inhibitor cocktail (Roche, Basel, Switzerland; Catalog No. 04693159001). Protein concentration was determined using a BCA kit (Thermo Fisher Scientific, Waltham, MA, USA; Catalog No. 23225). Proteins were transferred onto PVDF membranes (Millipore, Burlington, MA, USA; Catalog No. IPVH00010). Primary antibodies used: anti-COL3A1 (Abclonal, Wuhan, China; Catalog No. A1352, 1:1000), anti-β-actin (Abclonal, Wuhan, China; Catalog No. AC026, 1:2000). HRP-conjugated secondary antibodies (Abclonal, Wuhan, China; Catalog Nos. AS014 and AS003) were used. Immunoreactivity was detected using ECL substrate (Millipore; Catalog No. WBKLS0500).

### 2.17. Wound Healing Assay

Transfected cells were plated into six-well dishes, grown until nearly confluent, and subjected to straight scratch creation using a sterile pipette tip (200 µL). Wash steps with phosphate-buffered saline followed before maintenance in medium without serum. Phase-contrast photographs were recorded at initiation (0 h) and at 24 h employing an inverted microscope. Migratory efficiency was estimated by calculating the percentage of the original scratched zone filled within 24 h, according to the formula: percentage of wound healed = [(area at 0 h–area at 24 h) ÷ initial area] × 100.

### 2.18. Animal Grouping and Treatment

All experimental manipulations involving animals received approval from the Institutional Animal Care and Use Committee of Fudan University’s School of Pharmacy. Female NSG mice aged between four and six weeks were obtained from GemPharmatech (Nanjing, Jiangsu, China), and subsequently housed under strict pathogen-free conditions. A suspension of H1975 cells (5 × 10^6^) in PBS was administered via subcutaneous injection into the right flank of each subject. Tumor development was observed triennially by recording caliper measurements, with tumor volume estimation following the formula L × W^2/2^. When tumors reached approximately 50–100 mm^3^, mice received intravenous injection of human PBMCs (5 × 10^6^ cells per mouse) to establish an immune-reconstituted model. Animals were then randomly assigned to four groups: control (intratumoral negative control siRNA plus intraperitoneal vehicle), si-COL3A1 (intratumoral si-COL3A1 plus vehicle), anti-PD-L1 (BioXCell, Lebanon, NH, USA; Catalog No. BE0101, clone 10F.9G2) (control siRNA plus anti-PD-L1 antibody), and combination therapy (si-COL3A1 plus anti-PD-L1). siRNA (5 nmol per mouse) was administered intratumorally and anti-PD-L1 antibody (5 mg/kg) intraperitoneally twice per week for two weeks.

### 2.19. Statistical Analysis

All experimental datasets were subjected to two-sided Student’s *t*-test for the examination of variations between two different cohorts. In cases involving more than two groups, comparisons were conducted utilizing one-way ANOVA, complemented by suitable post hoc assessments to guarantee precision. Statistical significance throughout all analyses was established when the *p*-value was less than 0.05, while every test applied was distinctly two-sided.

GraphPad Prism 9.0 and R version 4.4.2 were employed for undertaking the statistical calculations presented within the study. For computational comparisons, such as evaluating pathway scores and differential gene expression, the Wilcoxon rank-sum test was selected unless other methods are specifically mentioned. The differences in Restricted Mean Survival Time (RMST) were assessed using the two-sided Z-test approach.

Survival distributions were estimated and tested using Kaplan–Meier curves with log-rank tests. Cox proportional hazards regression models provided estimations for hazard ratios (HRs) as well as their corresponding 95% confidence intervals.

## 3. Result

### 3.1. sc-RNA Profiling of Public NSCLC Datasets

To construct a comprehensive single-cell atlas of non-small cell lung cancer (NSCLC), scRNA-seq datasets were collected from multiple publicly available studies, including EMTAB6149 [29], EMTAB6653, GSE117570 [30], GSE123902 [31], GSE127465 [32], GSE131907 [33], GSE148071 [34], GSE148466 [35], GSE189357 [36], PRJNA591860, PRJNA634159 [37], and PRJNA698465 [38]. After quality control and data integration, a total of 650,461 cells derived from 216 samples (193 tumor and 23 normal tissues) were retained for downstream analyses.

**Figure 1:**
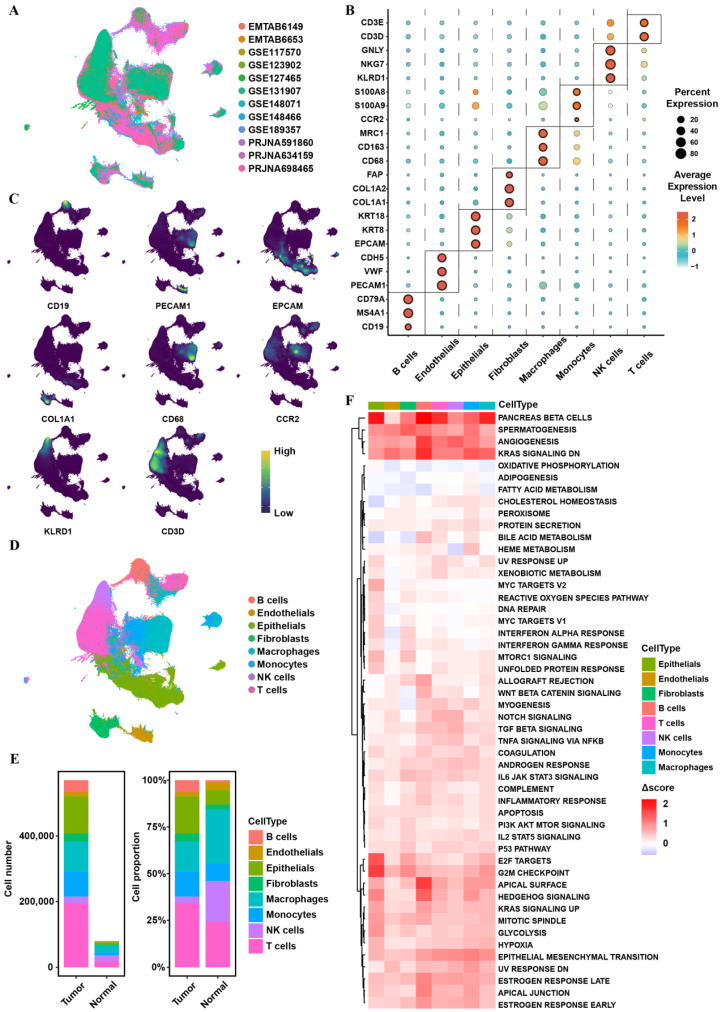
sc-RNA profiling of public NSCLC datasets. (**A**) UMAP visualization of integrated NSCLC single-cell data colored by dataset source. (**B**) DotPlot showing canonical marker gene expression across cell populations. (**C**) Distribution of representative marker genes projected onto the UMAP embedding. (**D**) Annotated UMAP plot showing major cell populations. (**E**) Cell numbers (left) and relative proportions (right) of major cell types in tumor and normal tissues. (**F**) Heatmap showing differences in Hallmark pathway activity between tumor and normal states across cell types (Δscore = Tumor − Normal).

UMAP visualization colored by dataset source demonstrated consistent cellular distributions across studies, indicating effective batch integration (Figure 1A). Canonical marker gene expression across major cell populations was visualized using DotPlot analysis (Figure 1B), and projection of representative markers onto the UMAP embedding further supported accurate cell-type annotation (Figure 1C). The annotated UMAP map revealed the major cellular components within the NSCLC tumor microenvironment (Figure 1D). Quantification of cell numbers and relative proportions highlighted compositional differences between tumor and normal tissues (Figure 1E). Functional comparison using ssGSEA showed widespread alterations in Hallmark pathway activities across cell types between tumor and normal states (Figure 1F). Tumor samples exhibited increased activation of pathways closely associated with cancer progression, including EMT, hypoxia response, glycolysis, and cell cycle-related programs (G2M checkpoint and E2F targets). In contrast, pathways related to metabolic homeostasis were relatively enriched in normal cells. Notably, pathway alterations displayed strong cell-type-specific patterns, highlighting functional heterogeneity within the NSCLC tumor microenvironment.

Together, these analyses established a high-quality integrated single-cell landscape of NSCLC and provided the foundation for dissecting tumor epithelial heterogeneity and identifying clinically relevant malignant programs.

### 3.2. Identification of an Aggressive Malignant Epithelial Cell State Associated with Poor Prognosis in NSCLC

**Figure 2:**
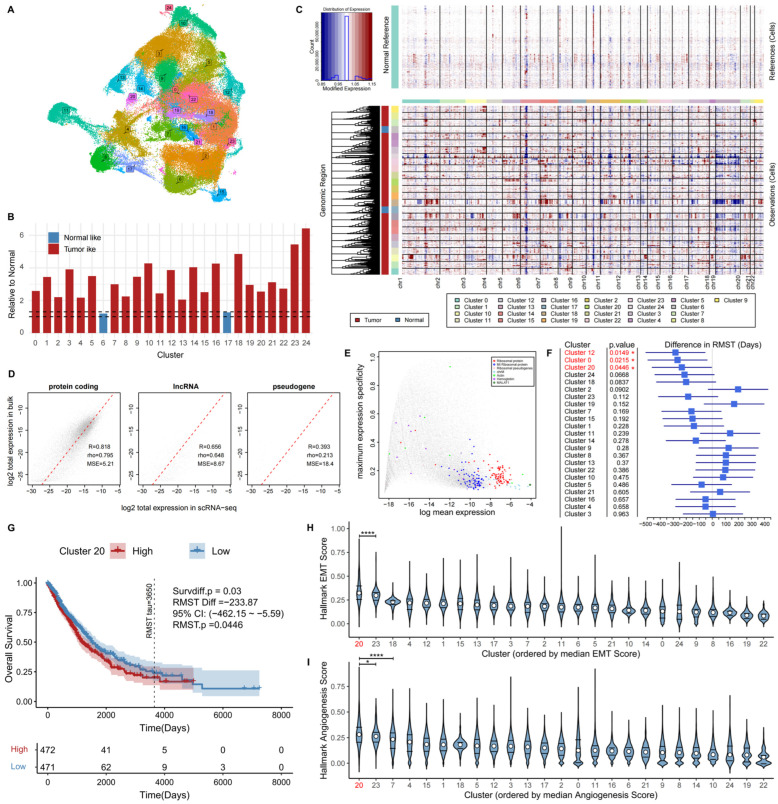
Identification of an aggressive malignant epithelial cell state associated with poor prognosis in NSCLC. (**A**) UMAP visualization showing reclustering of tumor-derived epithelial cells into 25 subpopulations, where different colors are used to distinguish individual clusters. (**B**) Inferred CNV profiles across epithelial subclusters using normal epithelial cells as reference. (**C**) Relative CNV scores distinguishing tumor-like and normal-like epithelial populations. (**D**) Correlation analysis demonstrating concordance between gene expression profiles derived from bulk RNA-seq and scRNA-seq data. (**E**) Removal of noise-associated genes during Bayesian deconvolution to improve signal specificity. (**F**) Forest plot showing associations between tumor subpopulation abundance and restricted mean survival time (RMST) (* *p* < 0.05). (**G**) Kaplan–Meier survival curves comparing patients with high versus low proportions of Cluster 20. (**H**,**I**) Comparative analysis of ssGSEA-derived pathway activity scores across different tumor cell subpopulations, ordered by descending median scores: (**H**) epithelial–mesenchymal transition (EMT); (**I**) angiogenesis. Pathway scores were calculated at the single-cell level using Hallmark gene sets from MSigDB by averaging the normalized expression of genes within each pathway and Student’s *t* test was used (* *p* < 0.05, **** *p* < 0.0001).

To resolve intratumoral epithelial heterogeneity, tumor-derived epithelial cells were reclustered, identifying 25 transcriptionally distinct subpopulations visualized by UMAP (Figure 2A). Copy number variation (CNV) inference using normal epithelial cells as reference revealed substantial genomic heterogeneity across clusters. Several subpopulations exhibited widespread chromosomal amplifications and deletions consistent with malignant transformation, whereas others displayed genomic stability comparable to normal epithelial cells (Figure 2B). Based on relative CNV scores, epithelial cells were categorized into tumor-like and normal-like populations, with Cluster 6 and Cluster 17 exhibiting normal-like genomic profiles and therefore excluded from subsequent tumor-specific analyses (Figure 2C).

To assess clinical relevance, bulk RNA-seq data from the TCGA-LUAD and TCGA-LUSC cohorts were deconvoluted using the BayesPrism algorithm to estimate patient-level abundance of each epithelial subpopulation. Removal of ribosomal and mitochondrial genes improved concordance between bulk and single-cell protein-coding gene expression profiles, while long non-coding RNAs and pseudogenes were largely excluded, indicating that the deconvolution captured biologically informative transcriptional programs (Figure 2D,E).

Survival analysis based on inferred subpopulation abundance identified several tumor epithelial clusters significantly associated with clinical outcomes (Figure 2F). This observation was further supported by ssGSEA analysis, which provided a global overview of pathway activity patterns across tumor epithelial subpopulations, highlighting distinct transcriptional profiles and facilitating the identification of functionally divergent clusters. Among these, Cluster 20 exhibited the most distinct pathway enrichment profile (Figure S1). Kaplan–Meier analysis confirmed reduced overall survival in patients enriched for Cluster 20, accompanied by a shortened RMST [difference = −233.87 days, 95% CI (−462.15 to −5.59), *p* = 0.0446] (Figure 2G). These findings suggest that the transcriptional program represented by Cluster 20, rather than overall epithelial content, contributes to adverse clinical outcomes.

Functional characterization further demonstrated that, compared with other prognostically associated clusters, Cluster 20 uniquely exhibited markedly increased EMT and angiogenesis pathway activities (Figure 2H,I). These transcriptional features are hallmarks of invasive and tumor-promoting phenotypes, indicating that Cluster 20 represents an aggressive malignant epithelial cell state.

Collectively, these findings define Cluster 20 as a clinically relevant high-risk malignant epithelial cell state in NSCLC, linking invasion- and angiogenesis-related transcriptional programs with poor patient survival.

### 3.3. Identification and Evolutionary Characterization of COL3A1^+^ Tumor Cells

**Figure 3:**
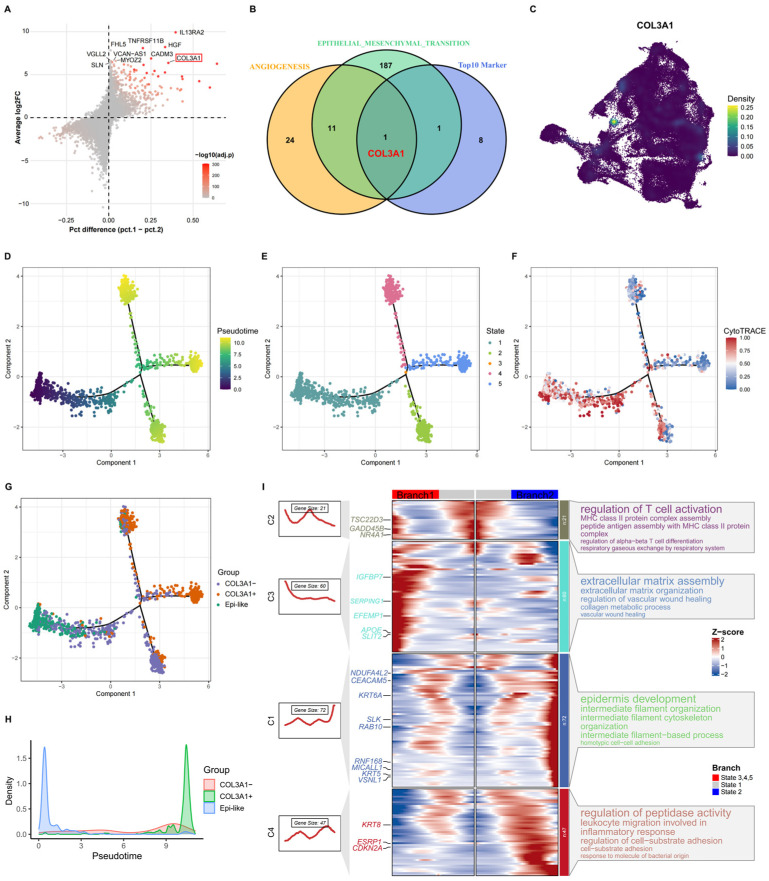
Identification and evolutionary characterization of COL3A1^+^ tumor cells. (**A**) Volcano plot of differentially expressed genes in Cluster 20. (**B**) Venn diagram showing overlap of top upregulated genes with EMT and angiogenesis pathways, highlighting COL3A1. (**C**) UMAP projection of COL3A1 expression across tumor cells. (**D**) Pseudotime trajectory of NSCLC tumor cells. (**E**) Distribution of cell states along pseudotime. (**F**) Comparison of differentiation scores across tumor cell populations. (**G**) Mapping of COL3A1^−^, COL3A1^+^, and epithelial-like cells along pseudotime. (**H**) Changes in relative abundance of different cell types along pseudotime. (**I**) Heatmap of branch-specific gene modules and functional enrichment identified by BEAM analysis.

To identify key molecular drivers underlying the aggressive phenotype of Cluster 20, we focused on the top upregulated genes and their pathway associations. The top 10 upregulated genes were intersected with Hallmark EMT and angiogenesis gene sets. COL3A1 emerged as the only gene shared by both pathways and displayed the highest expression specificity within Cluster 20 (Figure 3A–C), suggesting a potential role linking extracellular matrix remodeling with angiogenic activation. COL3A1 encodes the α1 chain of type III collagen, a major extracellular matrix component involved in tissue remodeling and tumor microenvironment regulation.

Pseudotime trajectory analysis revealed the evolutionary progression of tumor epithelial cells (Figure 3D,E). COL3A1^+^ tumor cells exhibited lower differentiation scores compared with COL3A1^−^ and epithelial-like populations (Figure 3F) and were predominantly localized at the terminal region of the trajectory (Figure 3G). Consistently, the relative abundance of COL3A1^+^ cells progressively increased along pseudotime, indicating enrichment during late tumor evolution (Figure 3H).

Branch-dependent gene expression analysis identified dynamic transcriptional programs along the COL3A1^+^ lineage. Genes enriched along this branch were associated with extracellular matrix organization, collagen metabolism, angiogenesis, and cell migration, whereas the alternative branch was characterized by epithelial maintenance and cell adhesion programs (Figure 3I).

These results indicate that COL3A1^+^ cells represent a late-stage, poorly differentiated tumor population undergoing functional reprogramming toward invasive and pro-malignant phenotypes. Given the established links between EMT, angiogenesis, and immune evasion programs, these findings raised the possibility that COL3A1 may also influence tumor-immune regulatory circuits.

### 3.4. Prognostic Evaluation and Model-Based Validation of COL3A1 in NSCLC

**Figure 4:**
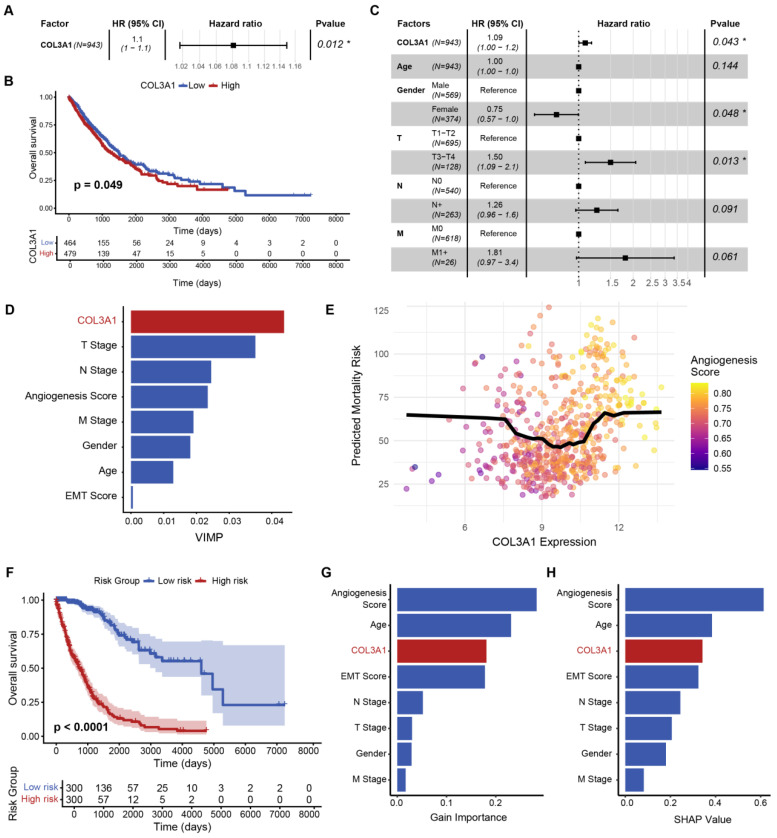
Prognostic evaluation and model-based validation of COL3A1 in NSCLC. (**A**) Forest plot showing hazard ratio (HR) and 95% confidence interval derived from univariate Cox regression analysis of COL3A1 expression (* *p* < 0.05). (**B**) Kaplan–Meier overall survival curves comparing patients with high and low COL3A1 expression. (**C**) Multivariate Cox regression analysis evaluating the independent prognostic value of COL3A1 after adjustment for clinical covariates (* *p* < 0.05). (**D**) Variable importance ranking derived from the random survival forest (RSF) model. (**E**) Partial dependence plot illustrating the nonlinear association between COL3A1 expression and predicted mortality risk. (**F**) Kaplan–Meier survival curves stratified by RSF-derived risk scores. (**G**,**H**) Feature importance ranking from the XGBoost survival model based on Gain and SHAP importance metrics.

To evaluate the clinical relevance of COL3A1 expression in NSCLC, survival analyses were conducted using TCGA cohorts. Univariate Cox regression demonstrated that elevated COL3A1 expression was significantly associated with poorer overall survival, with a hazard ratio greater than 1 (Figure 4A). Kaplan–Meier analysis further confirmed significantly shortened survival in patients with high COL3A1 expression (Figure 4B). Importantly, multivariate Cox regression incorporating age, sex, and TNM staging showed that COL3A1 remained a significant risk factor after adjustment (Figure 4C), indicating that its prognostic impact represents an independent molecular determinant beyond established clinicopathological variables.

To assess the robustness of this association under non-linear modeling frameworks, machine learning-based survival analyses were performed. In the RSF model, variable importance analysis consistently identified COL3A1 among the most informative predictors contributing to survival risk (Figure 4D). Partial dependence analysis revealed a nonlinear relationship between COL3A1 expression and predicted mortality risk, suggesting a dose-dependent prognostic effect across expression levels (Figure 4E). Stratification of patients according to RSF-derived risk scores yielded clearly separated survival curves, supporting the model’s discriminative capacity (Figure 4F).

To further evaluate the robustness of this association under an independent modeling framework, an XGBoost survival model was constructed. Feature importance rankings based on Gain and SHAP metrics consistently placed COL3A1 among the top contributing variables (Figure 4G,H). SHAP dependence analysis further demonstrated nonlinear and context-dependent risk contributions of COL3A1, with stronger effects observed at higher expression levels, particularly in samples with elevated angiogenesis pathway activity. These findings suggest that the prognostic effect of COL3A1 reflects complex interactions within the survival model rather than a simple linear relationship, providing additional SHAP-based evidence showing consistent and interpretable contributions of COL3A1 across samples, beyond global feature importance rankings (Figure S2).

Taken together, conventional survival analyses and machine learning models derived from distinct methodological frameworks consistently demonstrated a robust association between COL3A1 expression and adverse clinical outcomes in NSCLC. Beyond functioning as a prognostic marker, COL3A1 exhibits stable and context-dependent risk contributions across both mechanistically distinct models. In addition, its enrichment in aggressive malignant epithelial states identified at single-cell resolution further supports a functional role in tumor progression and highlights its potential as a biologically meaningful therapeutic target.

### 3.5. Simulated Knockout of COL3A1 Reveals Enrichment of PD-L1 Upstream Signaling Pathways

**Figure 5:**
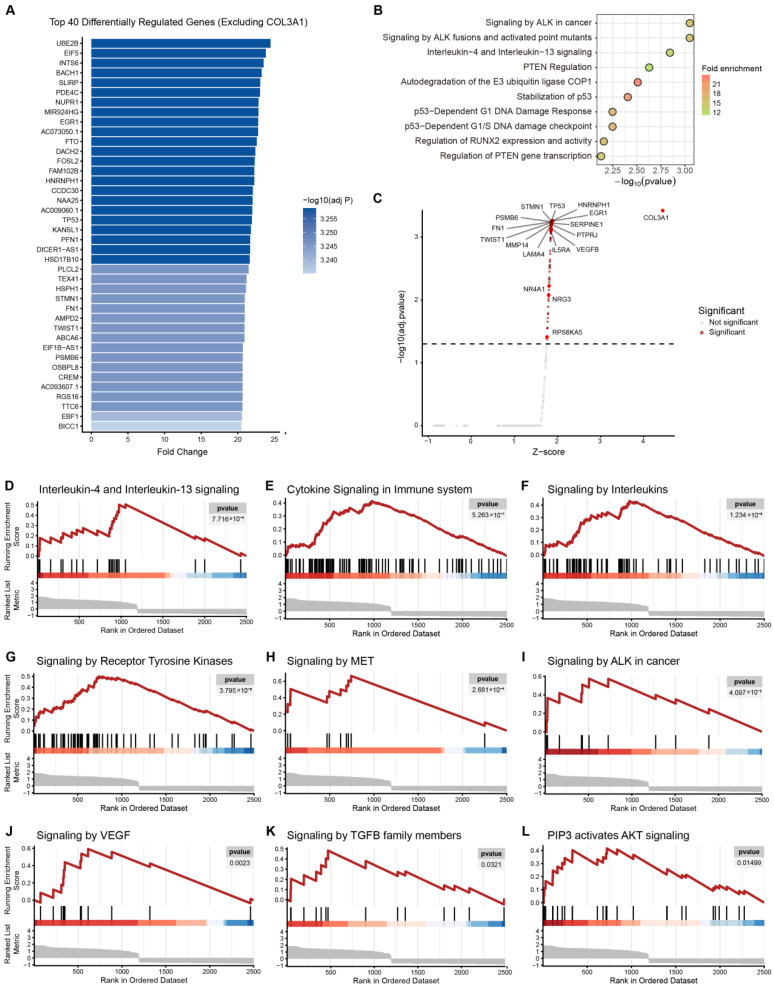
Simulated knockout of COL3A1 reveals PD-L1 upstream signaling perturbation. (**A**) Bar plot showing the top 40 differentially regulated genes ranked by perturbation strength following simulated knockout of COL3A1 in Cluster 20 tumor cells. (**B**) Reactome pathway enrichment analysis of the top 40 differentially regulated genes. (**C**) Volcano plot displaying global transcriptional changes after COL3A1 knockout, with PD-L1 upstream pathway-associated genes highlighted (adj.pvalue < 0.05). (**D**–**L**) GSEA showing enrichment of PD-L1 upstream signaling pathways.

To investigate potential regulatory functions of COL3A1 within tumor-intrinsic networks, we performed simulated knockout analysis using scTenifoldKnk in Cluster 20 tumor cells. As this analysis was performed specifically in malignant epithelial cells, the inferred regulatory changes reflect tumor-intrinsic transcriptional perturbations rather than direct effects from stromal or immune compartments. Comparison of inferred regulatory networks before and after perturbation identified widespread transcriptional changes. After excluding COL3A1 itself, the top 40 differentially regulated genes were ranked based on perturbation strength (Figure 5A). These genes included transcriptional regulators (EGR1, TP53, CREM) as well as genes involved in cell cycle control, stress response, and extracellular matrix remodeling (STMN1, FN1, TWIST1), indicating broad regulatory network rewiring following COL3A1 loss.

Reactome pathway enrichment analysis of the top 40 genes revealed significant enrichment of oncogenic and immune-related signaling pathways (Figure 5B). The most prominent pathways included Signaling by ALK in cancer and Interleukin-4 and Interleukin-13 signaling, together with multiple TP53-associated regulatory programs related to DNA damage response and cell-cycle control. Enrichment of RUNX2 regulatory signaling further suggested potential modulation of EMT-associated transcriptional programs, supporting a broad role of COL3A1 in tumor cell regulatory networks.

To globally assess transcriptional alterations, all differentially regulated genes were visualized using a volcano plot (Figure 5C). Several genes implicated in PD-L1 upstream regulation and immune-related signaling, including EGR1, TP53, FN1, TWIST1, and PSMB6, were significantly perturbed following COL3A1 knockout. In addition, angiogenesis- and microenvironment-associated genes (VEGFB, SERPINE1, MMP14, LAMA4) showed marked changes, suggesting coordinated remodeling of tumor-intrinsic signaling linked to immune regulation.

Gene set enrichment analysis (GSEA) further demonstrated significant enrichment of pathways known to regulate PD-L1 expression (Figure 5D–L). These included receptor tyrosine kinase signaling, cytokine signaling in the immune system, interleukin signaling, and multiple oncogenic pathways such as MET, VEGF, PI3K-AKT, and TGF-β signaling.

Collectively, these findings indicate that COL3A1 perturbation was associated with changes in multiple signaling pathways that are known to regulate PD-L1 expression, supporting a potential role of COL3A1 in shaping tumor-intrinsic transcriptional programs that may intersect with oncogenic and immune-related signaling pathways. These computational findings provided a mechanistic rationale for evaluating whether COL3A1 inhibition could enhance the therapeutic efficacy of PD-L1 blockade in vivo.

### 3.6. Functional Validation of COL3A1 Knockdown in NSCLC Cells

**Figure 6:**
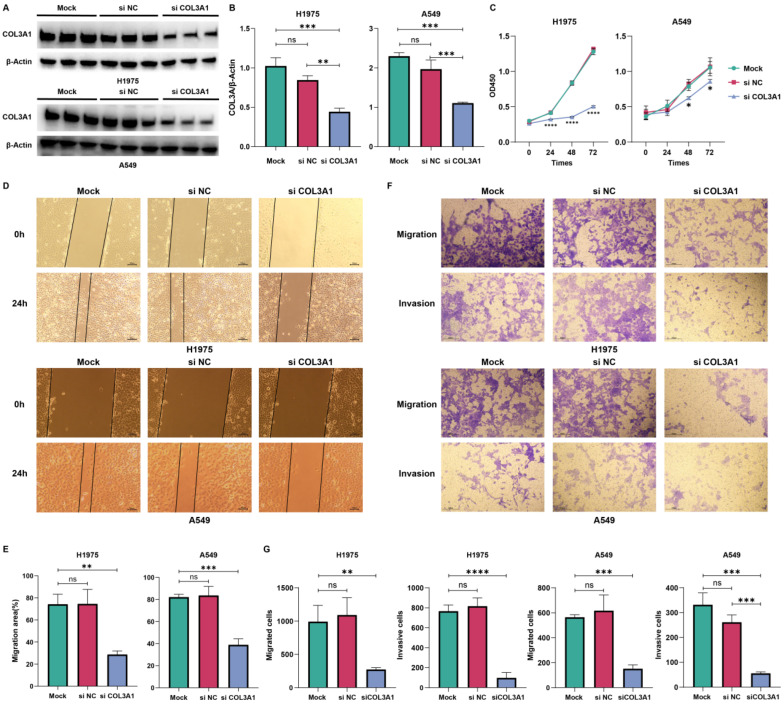
Functional validation of COL3A1 knockdown in NSCLC cells. (**A**) Knockdown of COL3A1 in H1975 and A549 cells was confirmed by Western blotting after transfection with siCOL3A1 or control siRNA (siNC), with β-actin used as the loading control for normalization. (**B**) Quantification of COL3A1 protein levels normalized to β-actin. (**C**) Cell proliferation was assessed by CCK-8 assays in H1975 and A549 cells at indicated time points after COL3A1 knockdown. (**D**) Representative images were captured at 0 h and 24 h post-scratch during wound healing assays of H1975 and A549 cells. (**E**) Quantification of wound closure rates expressed as percentage of closure. (**F**) Representative images of Transwell migration and invasion assays following COL3A1 knockdown. (**G**) Quantification of migrated and invaded cells. We performed statistical analyses using one-way ANOVA followed by Tukey’s multiple comparisons test unless otherwise indicated, and data are presented as mean ± SD. Student’s *t* test was used in (**C**) (ns, not significant; * *p* < 0.05, ** *p* < 0.01, *** *p* < 0.001, **** *p* < 0.0001).

To experimentally validate the functional role of COL3A1, H1975 and A549 NSCLC cells were subjected to siRNA-mediated knockdown of COL3A1. COL3A1 knockdown was achieved by siCOL3A1 transfection, as evidenced by Western blot analysis showing markedly reduced protein levels compared with Mock and siNC controls, while β-actin expression remained unchanged (Figure 6A). Quantification confirmed significant suppression of COL3A1 expression in both cell lines (Figure 6B), indicating efficient knockdown suitable for downstream functional analyses.

CCK-8 assays revealed that the largely overlapping growth curves of Mock and siNC groups indicated minimal non-specific effects of control siRNA treatment on cell proliferation. In contrast, COL3A1 knockdown significantly reduced cell proliferation in H1975 cells beginning at 24 h, with pronounced inhibition observed at later time points. A similar but more gradual reduction in proliferative capacity was observed in A549 cells (Figure 6C). These results indicate that COL3A1 promotes NSCLC cell growth.

Wound healing and Transwell assays were performed to evaluate the effect of COL3A1 depletion on cell motility. Control cells displayed substantial scratch closure after 24 h, whereas siCOL3A1-treated cells exhibited markedly impaired migration in H1975 and A549 cell lines in wound healing assays (Figure 6D,E). Consistently, Transwell assays revealed significantly reduced numbers of migrated and invaded cells following COL3A1 knockdown (Figure 6F,G), indicating impaired migratory and invasive capacities.

Collectively, these findings demonstrate that COL3A1 contributes to proliferative and invasive phenotypes of NSCLC cells, supporting its tumor-intrinsic role in promoting malignant progression, and providing experimental evidence for subsequent in vivo evaluation.

### 3.7. Combined COL3A1 Silencing and Anti-PD-L1 Treatment Enhanced Antitumor Efficacy In Vivo

**Figure 7:**
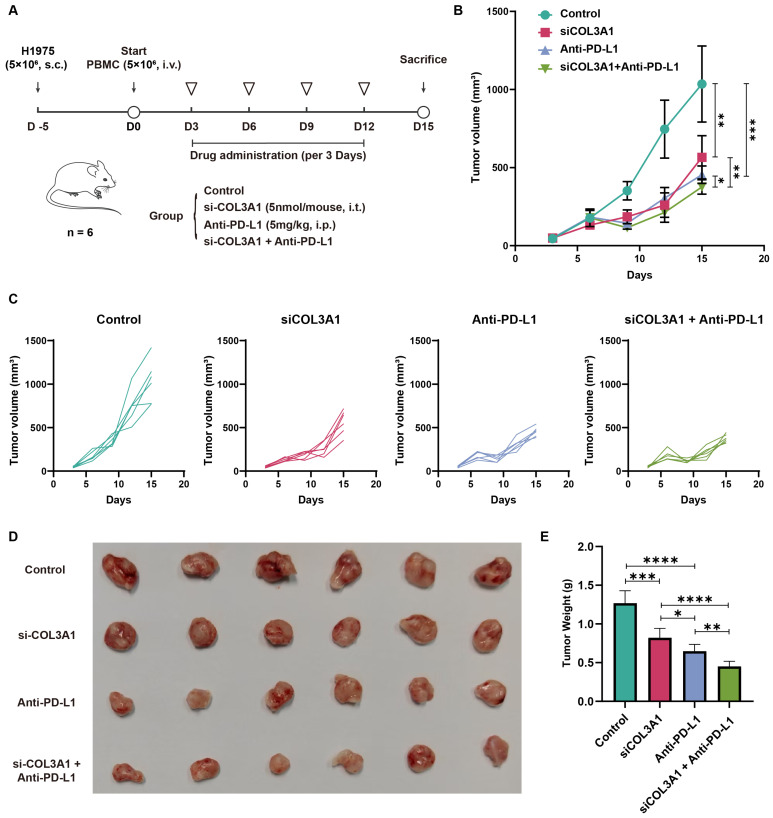
Combined COL3A1 silencing and Anti-PD-L1 treatment enhanced antitumor efficacy in vivo. (**A**) Schematic diagram depicting the humanized xenograft model and treatment schedule. (**B**) Tumor growth curves in control, siCOL3A1, Anti-PD-L1, and siCOL3A1 + Anti-PD-L1 groups. (**C**) Individual tumor growth trajectories for each mouse. (**D**) Excised tumor images were recorded at endpoint. (**E**) All data are shown as mean ± SD, with statistical significance assessed via one-way ANOVA and subsequent Student’s *t* test. (* *p* < 0.05, ** *p* < 0.01, *** *p* < 0.001, **** *p* < 0.0001).

To evaluate the therapeutic potential of targeting COL3A1 in vivo, a humanized xenograft model was established by intravenous injection of PBMCs followed by subcutaneous implantation of H1975 cells (Figure 7A). We randomly divided the mice into four groups (n = 6): Control, siCOL3A1, Anti-PD-L1, and siCOL3A1 + Anti-PD-L1.

Tumor growth was monitored over the course of treatment. At the endpoint (D15), both siCOL3A1 and anti–PD-L1 monotherapy showed significantly reduced tumor volumes compared with the control group (Figure 7B). Remarkably, the combination treatment yielded the strongest tumor growth inhibition, showing significantly smaller tumor volumes at endpoint compared with either single-treatment group.

Individual tumor growth curves further demonstrated consistent reduction in tumor progression across mice receiving combination therapy (Figure 7C). At sacrifice, excised tumors in the combination group were visibly smaller than tumors in control or monotherapy groups (Figure 7D). Quantification of tumor weights confirmed significant reductions in the siCOL3A1 and Anti-PD-L1 groups, with the greatest decrease observed in the combination group (Figure 7E).

Collectively, these findings demonstrate that COL3A1 silencing enhances the antitumor therapeutic effect of Anti-PD-L1 in vivo, supporting a functional interaction between tumor-intrinsic COL3A1 signaling and immune checkpoint blockade.

## 4. Discussion

In this study, we integrated large-scale single-cell and bulk transcriptomic datasets to systematically characterize malignant epithelial heterogeneity in NSCLC and identify tumor-intrinsic drivers of aggressive disease states. Our analyses revealed a distinct epithelial subpopulation enriched for EMT and angiogenesis programs and strongly associated with poor clinical outcomes. Within this lineage, COL3A1 emerged as a key molecule associated with extracellular matrix remodeling and aggressive tumor states, and may be linked to immune-related transcriptional programs. By integrating CNV inference and Bayesian deconvolution of TCGA cohorts, we found that patient prognosis was associated with the abundance of specific malignant epithelial states rather than the overall proportion of epithelial cells. Our results indicate that tumor outcomes are more closely associated with the composition of malignant epithelial states than with overall epithelial abundance. The enrichment of Cluster 20 identifies a particularly aggressive transcriptional program that contributes to poor prognosis. Trajectory analysis further positioned COL3A1^+^ tumor cells at a late-stage, poorly differentiated branch characterized by increased invasive and angiogenic features. Type III collagen, encoded by COL3A1, is a major extracellular matrix component traditionally associated with stromal remodeling. Our findings extend this paradigm by suggesting a tumor-intrinsic role for COL3A1 within malignant epithelial cells themselves. The enrichment of COL3A1 in advanced pseudotime states suggests its association with tumor progression and phenotypic plasticity. Importantly, survival analyses across conventional Cox regression and mechanistically distinct machine learning frameworks consistently demonstrated the independent prognostic value of COL3A1. The nonlinear and context-dependent risk contributions observed in RSF and XGBoost models suggest that COL3A1 expression may interact with broader oncogenic signaling networks rather than acting as a simple linear biomarker. Virtual knockout analysis revealed that COL3A1 perturbation coordinately affects multiple oncogenic and cytokine pathways implicated in PD-L1 regulation, including RTK, PI3K-AKT, TGF-β, and interleukin signaling. These findings suggest a potential association between tumor-intrinsic extracellular matrix remodeling and transcriptional programs related to immune checkpoint regulation. Consistent with this hypothesis, combined COL3A1 silencing and PD-L1 blockade demonstrated superior antitumor efficacy in vivo compared with monotherapy. This synergy supports the notion that targeting tumor-intrinsic regulators may enhance immunotherapy responsiveness.

Several limitations of this study should be acknowledged. First, functional validation was primarily achieved through transient knockdown of COL3A1, and no stable inhibitory strategy, such as antibodies or small molecules, was employed. Future studies are warranted to explore clinically applicable approaches for sustained COL3A1 inhibition and to evaluate their therapeutic potential. Second, although our computational perturbation and combination treatment experiments suggest a functional interaction between COL3A1 signaling and PD-L1-related pathways, the precise molecular mechanisms underlying this regulation remain to be elucidated. Detailed mechanistic studies will be required to define whether COL3A1 modulates immune checkpoint expression or acts through intermediary oncogenic signaling cascades. Third, this study relied on publicly available single-cell and bulk transcriptomic datasets. While large-scale integration enhances statistical power and generalizability, prospective validation using independent patient-derived sequencing cohorts would further strengthen the translational relevance of our findings. Finally, in vivo experiments were conducted using a humanized xenograft model, which may not fully recapitulate the complexity and heterogeneity of human immune responses. More physiologically representative preclinical models and eventual clinical validation will be necessary to confirm the therapeutic potential of COL3A1 targeting in combination with PD-L1 blockade.

Despite these limitations, our multi-level integrative framework provides a comprehensive view of tumor-intrinsic extracellular matrix regulation in NSCLC and lays the groundwork for rational combination immunotherapy strategies. These results support the role of epithelial cell state heterogeneity in shaping tumor progression and suggest that extracellular matrix-associated programs may contribute to malignant phenotype diversity and therapeutic response.

## 5. Conclusions

In this study, we integrated single-cell and bulk transcriptomic analyses to characterize malignant epithelial heterogeneity in NSCLC. We identified a distinct EMT- and angiogenesis-associated epithelial cell state that is linked to poor prognosis. Within this state, COL3A1 was identified as a tumor-intrinsic gene associated with aggressive malignant phenotypes and extracellular matrix remodeling. Functional and computational analyses suggested that COL3A1 may be linked to tumor-intrinsic signaling networks associated with immune checkpoint pathways. Importantly, combined inhibition of COL3A1 and PD-L1 blockade enhanced antitumor efficacy in vivo. These findings highlight COL3A1 as a potential therapeutic target and support the concept that targeting tumor cell states may improve immunotherapy outcomes in NSCLC.

## Data Availability

The original contributions presented in this study are included in the article/Supplementary Material. Further inquiries can be directed to the corresponding author(s).

## Supplementary Materials

The following supporting information can be downloaded at: https://www.mdpi.com/article/10.3390/biomedicines14050975/s1, Figure S1: Heatmap showing Hallmark pathway activity scores (ssGSEA) across tumor-derived epithelial cell subpopulations in the integrated NSCLC single-cell atlas. Cluster 20 displays the most distinct enrichment pattern, highlighting elevated EMT and angiogenesis programs compared with other subpopulations. Figure S2: SHAP dependence plot for COL3A1 in the XGBoost survival model. The plot illustrates the nonlinear association between COL3A1 expression and predicted mortality risk, with marginal effects shown to be context-dependent. Points are colored by angiogenesis ssGSEA scores, highlighting that at extremely high COL3A1 expression, the model assigns relatively lower risk contribution when angiogenesis signaling is already strongly activated, reflecting the context-specific nature of COL3A1’s prognostic effect.
