# Supplementary material for: Integrative Transcriptomic Analysis Identifies COL3A1 as a Potential Tumor-Intrinsic Therapeutic Target in NSCLC
Source: Biomedicines. 2026 Apr 23;14(5):975. doi: 10.3390/biomedicines14050975 (PMC13203928; doi:10.3390/biomedicines14050975)
Supplement: Supplementary file 1 [file biomedicines-14-00975-s001.zip › biomedicines-4238875-supplementary.pdf]

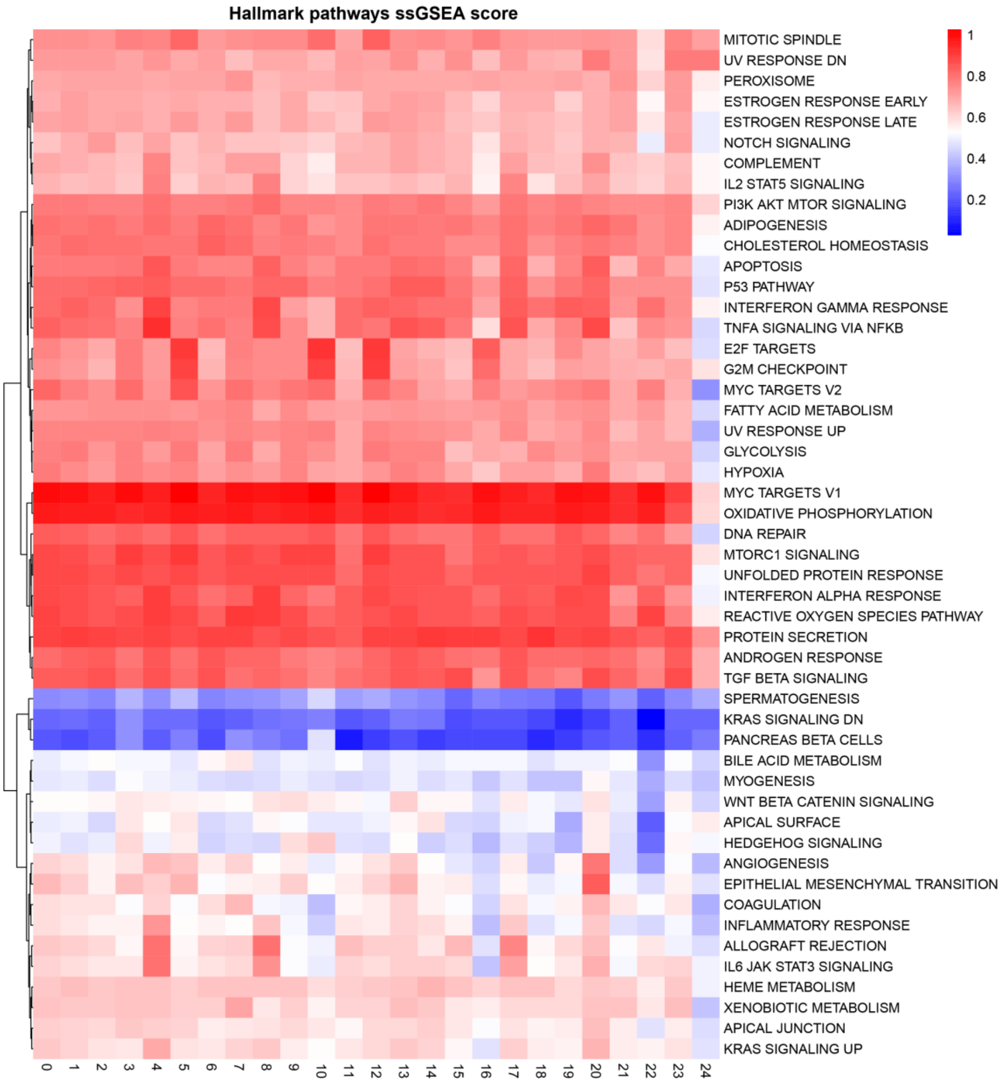

Figure S1. Heatmap showing Hallmark pathway activity scores (ssGSEA) across tumor-derived epithelial cell subpopulations in the integrated NSCLC single-cell atlas. Cluster 20 displays the most distinct enrichment pattern, highlighting elevated EMT and angiogenesis programs compared with other subpopulations.

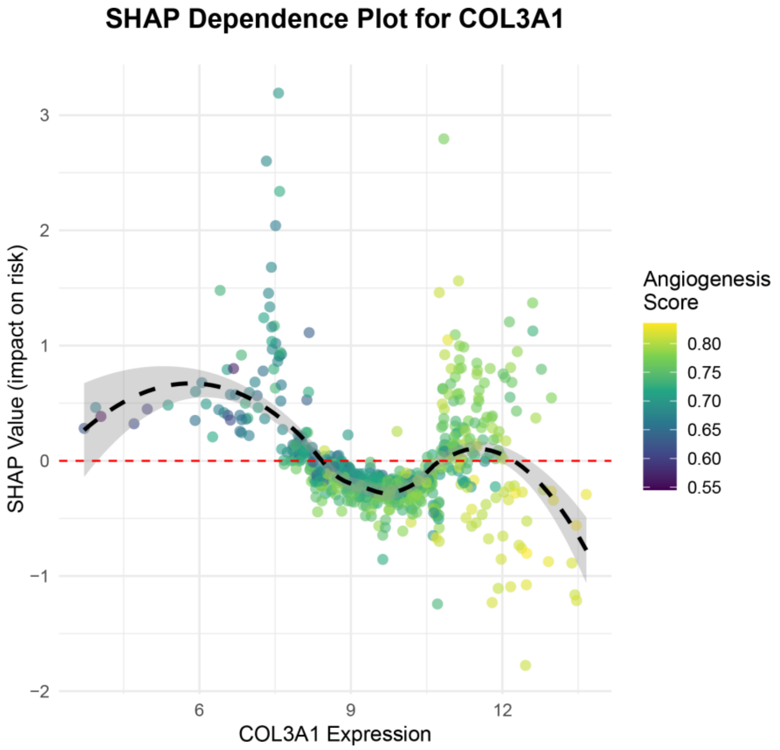

Figure S2. SHAP dependence plot for COL3A1 in the XGBoost survival model. The plot illustrates the nonlinear association between COL3A1 expression and predicted mortality risk, with marginal effects shown to be context-dependent. Points are colored by angiogenesis ssGSEA scores, highlighting that at extremely high COL3A1 expression, the model assigns relatively lower risk contribution when angiogenesis signaling is already strongly activated, reflecting the context-specific nature of COL3A1's prognostic effect.
